# The Performance of GRAMM-SCI and WRF in Simulating the Surface-Energy Budget and Thermally Driven Winds in an Alpine Valley

**DOI:** 10.1007/s10546-023-00835-9

**Published:** 2023-11-10

**Authors:** Gaspard Simonet, Dietmar Oettl, Manuela Lehner

**Affiliations:** 1 Department of Atmospheric and Cryospheric Sciences, University of Innsbruck, Innrain 52f, 6020 Innsbruck, Austria; 2 Air Quality Control, Regional Government of Styria, Landhausgasse 7, 8010 Graz, Austria

**Keywords:** Complex terrain, Land-atmosphere exchange, Model evaluation, Valley winds

## Abstract

Using WRF as a benchmark, GRAMM-SCI simulations are performed for a case study of thermally driven valley- and slope winds in the Inn Valley, Austria. A clear-sky, synoptically undisturbed day was selected when large spatial heterogeneities occur in the components of the surface-energy budget driven by local terrain and land-use characteristics. The models are evaluated mainly against observations from four eddy-covariance stations in the valley. While both models are able to capture the main characteristics of the surface-energy budget and the locally driven wind field, a few overall deficiencies are identified: (i) Since the surface-energy budget is closed in the models, whereas large residuals are observed, the models generally tend to overestimate the daytime sensible and latent heat fluxes. (ii) The partitioning of the available energy into sensible and latent heat fluxes remains relatively constant in the simulations, whereas the observed Bowen ratio decreases continuously throughout the day because of a temporal shift between the maxima in sensible and latent heat fluxes, which is not captured by the models. (iii) The comparison between model results and observations is hampered by differences between the real land use and the vegetation type in the model. Recent modifications of the land-surface scheme in GRAMM-SCI improve the representation of nighttime katabatic winds over forested areas, reducing the modeled wind speeds to more realistic values.

## Introduction

Air quality assessments in complex topography, such as the densely populated Alpine regions of Europe, require accurate flow fields with a high spatial resolution. The spatial variability of surface winds at length scales of 1 km or less can be large and depends strongly on the orography, but also on variations in land use. Under synoptically undisturbed conditions, thermally driven circulations form regularly in mountainous terrain, including up- and downslope flows along mountain sidewalls and up- and down-valley winds along the valley axes, which are driven by local temperature gradients resulting from spatial variations in solar heating of the valley atmosphere (Zardi and Whiteman 2012). Solar radiation depends strongly on the local slope angle and orientation of the surface, as well as on shadows cast by the surrounding topography (Whiteman et al. 1989; Matzinger et al. 2003; Hoch and Whiteman 2010; Lehner et al. 2021). These studies have shown that, while the incoming solar radiation peaks around solar noon on the approximately flat surface of a valley floor, diurnal cycles can become strongly asymmetric on the west- and east-facing slopes of a north–south oriented valley, with the maxima shifted to the morning and afternoon, respectively (Whiteman et al. 1989; Matzinger et al. 2003; Hoch and Whiteman 2010). In east–west-oriented valleys, such as the Inn Valley, Austria, which is the focus of this study, spatial variations in incoming solar radiation are, however, not as pronounced (Lehner et al. 2021). Diurnal cycles of sensible and latent heat fluxes between the surface and the atmosphere are strongly determined by the diurnal cycles in net radiation and thus solar incoming radiation. Whiteman et al. (1989) and Rotach and Zardi (2007) have shown for north–south-oriented valleys that the peak in sensible heat flux shifts from the morning on an east-facing sidewall over noon on a valley floor to the afternoon on a west-facing sidewall, similar to radiation. In addition to slope angle and orientation, land-use characteristics impact surface albedo and the partitioning of the available energy into sensible and latent heat fluxes and the diurnal cycles and the timing of the peaks can be further influenced by local flow circulations, as has been shown for the Inn Valley by Lehner et al. (2021).

Overall, most model evaluation studies are restricted to readily available quantities such as specific humidity, radiation, wind, and temperature (Tomasi et al. 2017; Jiménez-Esteve et al. 2018). A number of studies have evaluated the performance of mesoscale models in simulating flow fields in the Alps (Giovannini et al. 2014; Gsella et al. 2014; Cantelli et al. 2017; Schmidli et al. 2018; Schlager et al. 2019; Oettl 2021), but comparisons with observations of all surface-energy fluxes are still scarce in the scientific literature (one exception being Sun et al. 2017), especially in mountainous terrain and with respect to small-scale spatial variations. The above studies have highlighted some challenges in simulating the near-surface atmosphere over mountainous terrain. The results presented by Gsella et al. (2014), for example, have shown that model performance is higher for sites over plains and hilly terrain than for valley and mountain-top locations. While adequate horizontal model grid spacing is crucial to resolve small-scale wind structures (Jiménez-Esteve et al. 2018; Schmidli et al. 2018; Schlager et al. 2019; Oettl 2021), processes impacting the surface-energy budget components, such as topographic shading (Schlager et al. 2019), surface albedo (Tomasi et al. 2017), and land-use class (Jiménez-Esteve et al. 2018), have equally been identified as strongly impacting the modeled near-surface temperature and wind fields. Understanding model deficiencies in reproducing observed surface radiation, sensible, and latent heat fluxes over mountainous terrain is thus paramount to further improve numerical simulations.

In this work use was made of several flux tower observations in the Inn Valley, Austria, to evaluate the Graz Mesoscale Model-Scientific (GRAMM-SCI; Oettl 2023) and compare its performance with that of the state-of-the-art Weather and Research Forecasting model (WRF; Skamarock et al. 2008). Section 2 briefly introduces the two models and the respective model setups. The area of interest, the observational data, and the selected case study are described in Sect. 3. The model evaluation focuses in particular on the models’ ability to capture observed spatial variations in the surface-energy fluxes (Sects. 4.1–4.3). In addition, the evolution of the valley-wind circulation is compared between the two models and against upper-air observations (Sects. 4.5 and 4.6). Finally, a discussion and conclusions are given in Sects. 5 and 6, respectively.

## Numerical Models

### Graz Mesoscale Model-Scientific (GRAMM-SCI)

GRAMM-SCI is a new branch of the public and open source model GRAMM version 20.01 which is a non-hydrostatic model that solves the governing equations on a horizontal tetrahedron and on a vertically terrain-following coordinate system (Oettl 2021). Since 2019, GRAMM has been further developed by the Air Quality Control unit of Styria, Austria, to use ERA5 reanalysis data^1^ for initialization and for prescribing transient boundary conditions. The motivation for the development of GRAMM-SCI stems from the need for high-resolution (grid spacing $\le 1$ km) wind fields for air quality assessments, which can be produced at comparatively low computational costs (Oettl 2021). The model description and previous evaluation studies for Alpine regions have been published by Oettl (2020, 2021); Oettl and Veratti (2021); Oettl and Reifeltshammer (2023). In the following, model details and parameterizations relevant for this study will be briefly outlined.

**Fig. 1:**
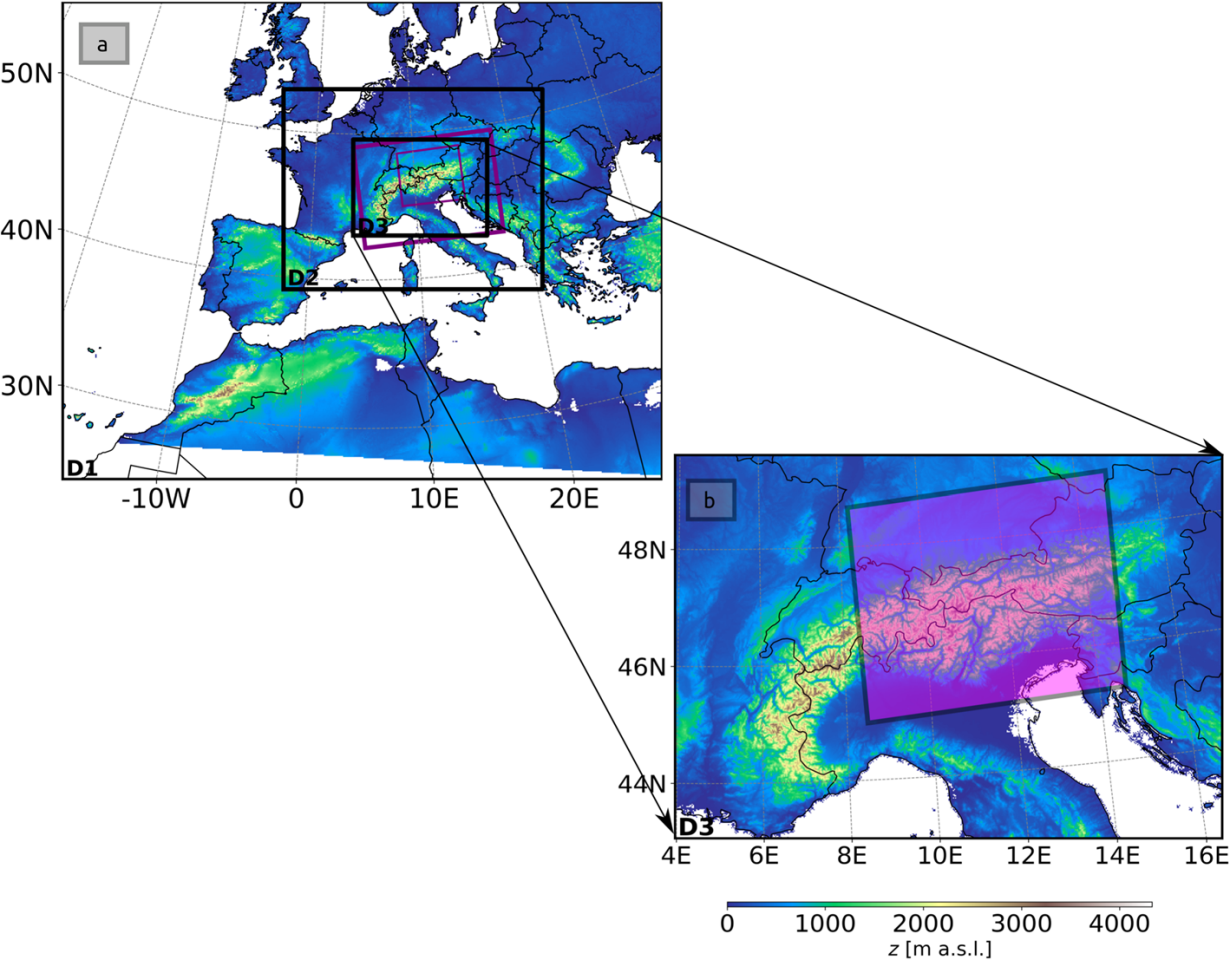
**a** Locations of the three WRF model domains depicted as black rectangles, with 9-km (D1), 3-km (D2), and 1-km (D3) horizontal grid spacing. The two innermost GRAMM-SCI domains with 4-km and 1-km horizontal grid spacing are depicted as purple rectangles. **b** D3 with the GRAMM-SCI model domain with a 1-km grid spacing highlighted by the purple rectangle

Three nested model domains with a horizontal grid spacing of 16 km, 4 km, and 1 km are used in this study following previous GRAMM-SCI simulations for Alpine regions (Oettl 2021; Oettl and Bergamin 2022; Giovannini et al. 2023). Note that the grid spacing of the innermost domain has been chosen to correspond to the resolution of current operational limited area models. ERA5 data on pressure levels with a 1-h interval are used to update the boundary conditions and for computing large-scale pressure gradients (Oettl 2021). 56 layers were defined in the vertical direction with 33 of them in the lowest 3 km and the first grid point 10 m above the surface. The model top was set to 18 km and the innermost model domain covered western Austria, while the outermost domains are almost identical with domain D3 of the WRF simulation (Fig. 1).

GRAMM-SCI uses the European Corine Land Cover (CLC18) dataset from the year 2018 provided by the Copernicus data service^2^ with a spatial resolution of 100 m. If the model resolution is coarser than 100 m and more than one land-use class is present within a model grid cell, GRAMM-SCI uses the median land-use parameters (e.g., albedo and roughness length) instead of the mean over all represented land-use classes. Radiation is calculated following Somieski (1988) and the surface fluxes depend on stability and roughness-length following the flux relations proposed by Businger et al. (1971). No microphysics scheme is implemented in GRAMM-SCI. Cloud and also snow cover are retrieved from ERA5 reanalysis data and are used diagnostically for adjusting the soil heat flux, albedo, and radiation (Oettl 2021).

It has already been stated in a previous evaluation study that GRAMM-SCI has a tendency to overestimate the velocities of katabatic slope winds (Oettl and Veratti 2021). The present work suggests that this deficiency is linked to the impact of forested areas on the sensible heat flux. To improve the model performance, the following three changes have been implemented for grid cells with forest cover, which have been previously tested for simulations over the Swiss Alps (Oettl and Bergamin 2022).

First, a drag law is introduced in the conservation equation for the wind components according to:

1 $$\begin{aligned} \left( \frac{\partial u_i}{\partial t}\right) _D = -C_D\ n\ \text {LAD}\ |U|\ u_i, \end{aligned}$$

where $C_D$ is an empirical drag coefficient ($0.15n^2$), *n* is the dimensionless vegetation coverage, LAD is the leave-area density [m$^{2}$ m$^{-3}$], $u_i$ is the wind component in direction *i* [m s$^{-1}$], and |*U*| is the total wind speed [m s$^{-1}$]. The above drag law is only applied at grid cells containing a forest based on the tree height, which is assumed to be 10 m for agro-forests and 20 m for all other types. Depending on the thickness of the first model layer, the drag law is thus typically applied at the lowermost one or two model levels. Fixed values are used for LAD with 0.1 m$^{2}$ m$^{-3}$ for deciduous and agro-forests and 0.15 m$^{2}$ m$^{-3}$ for coniferous forests. The same approach has already been used in previous studies by Wagner et al. (2019) and Leukauf et al. (2019) using WRF.

Second, as pointed out by Stuenzi et al. (2021), forests have a large impact on the radiative and turbulent heat fluxes. One of the main sources for increased surface temperatures within the forest canopy during the night is the so-called below-canopy longwave enhancement. This effect has been accounted for in GRAMM-SCI in a simplified way by reducing the emissivity $\epsilon$ of forested areas. In the current version, the following values have been assigned: $\epsilon = 0.6$ for coniferous forests (throughout the year) and deciduous forests (throughout the year in tropical and subtropical regions and during summer in mid-latitude regions) and $\epsilon = 0.7$ for deciduous forests in mid-latitudes during winter. The fraction of forest cover and the partitioning into different types of forest is taken into account in calculating the overall emissivity of each grid cell.

Third, the roughness length is limited to $z_0\le 0.1$ m in the calculation of the surface-atmosphere heat exchange to avoid excessive heat fluxes over rough terrain, similar to the Consortium for Small-scale Modeling model (COSMO; Doms et al. 2011).

GRAMM-SCI has no full land-surface model and the water budget equation is not parameterized. The deep-soil temperature and soil moisture, which are defined at 1.636 m below the surface, are updated from ERA5 data every six hours, while the soil temperatures above are calculated in eight soil layers, which are located at depths of 0.004, 0.013, 0.027, 0.06, 0.136, 0.311, 0.712, and 1.636 m.

In the former GRAMM-SCI version, turbulent mixing was represented through a simple first order turbulence model based on Pandolfo (1971), computing the eddy viscosity through the mixing length and a Richardson-number dependent coefficient (see Oettl 2023, for details). In the current version, a 1.5-order turbulence closure solving the prognostic equation for the turbulent kinetic energy *k* has been introduced.

According to Launder and Spalding (1983) the turbulent exchange coefficient can be expressed as $\mu _t = 0.09\dfrac{k^2}{\epsilon _k}$. Bougeault and Lacarrere (1989) suggested $\epsilon _k = \dfrac{C_{\epsilon ~k^{1.5}}}{l_{\epsilon }}$ which results in $\mu _t = 0.09\dfrac{\sqrt{k}~l_{\epsilon }}{C_{\epsilon }}$. Further, Bougeault and Lacarrere (1989) proposed for the mixing length of the dissipation rate $l_{\epsilon } = \sqrt{l_{up}~l_{down}}$. In GRAMM-SCI $l_{up}$ is set equal to the height above ground *z*, and $l_{down}$ equal to the difference between the height above ground and the mixing height of the boundary layer $z_l$, giving $\mu _t = 0.09\dfrac{\sqrt{z(z_l-z)}~\sqrt{k}}{C_{\epsilon }}$ where the mixing length $z_l$ is estimated using the empirical relationships proposed by Hanna (1982). While Bougeault and Lacarrere (1989) proposed $\dfrac{1}{C_{\epsilon }} = 1.4$, in GRAMM-SCI a value of 1.0 is used instead. This choice is based on various model evaluation studies and it has already been applied within the TEAMx model intercomparison study (Giovannini et al. 2023) and within a modelling study for the Swiss Alps (Oettl and Bergamin 2022). In the surface layer, which is equivalent to the first model layer, the turbulent kinetic energy and dissipation rate are computed as:

2 $$\begin{aligned} k=\dfrac{u^2_*}{\sqrt{C_\mu }}, \end{aligned}$$

and:

3 $$\begin{aligned} \epsilon _k=\dfrac{u^3_*}{k~z}, \end{aligned}$$

respectively.

To compare the model output with data measured at heights lower than the first model level at 10 m a.g.l., the potential temperature at sensor height $\theta _s$ is calculated using similarity theory analogous to the formulation in the Atmospheric Research Mesoscale Model (MM5) surface-layer scheme (Grell et al. 1994):

4 $$\begin{aligned} \theta _s = \theta _g + (\theta _{z_1}-\theta _g) \frac{ln\left( \dfrac{2}{z_0}\right) - \varPsi _h\left( \dfrac{2}{L}\right) }{ln\left( \dfrac{z_1}{z_0}\right) -\varPsi _h\left( \dfrac{z_1}{L}\right) }, \end{aligned}$$

where $z_1$ is the height of the lowest model level above ground, $z_0$ the roughness length, $\theta _g$ and $\theta _{z_1}$ the potential temperature at the surface and the first model level, respectively, *L* the Obukhov length, and $\varPsi _h$ the similarity function for heat according to Businger et al. (1971). For all measurements higher than the first grid point, a linear interpolation between the model levels is used to derive the quantities at the sensor heights.

### Weather Research and Forecasting Model (WRF)

WRF V4.0 and more specifically the Advanced Research core (ARW) is a state-of-the-art atmospheric research model developed by the National Center for Atmospheric Research (NCAR, Boulder, CO, USA). WRF allows the user to select from a large variety of physics parameterizations (Skamarock et al. 2008, 2019) and can be used to simulate atmospheric processes at scales ranging from meters to the entire globe. It is a fully compressible, non-hydrostatic model that solves the Euler equations in a hybrid vertical coordinate system, with terrain-following coordinates near the surface transitioning to isobaric coordinates aloft.

The WRF simulation for the present study was performed using three one-way nested domains, with the outermost domain D1 covering Europe and the innermost domain D3 spanning the entire Alps (Fig. 1). D1, D2 and D3 have 502$\times$400, 642$\times$502, 1003$\times$721 grid points and horizontal grid spacings of 9, 3 and 1 km, respectively. To ensure model stability and following Umek et al. (2021, 2022) who also used WRF over the Inn Valley to simulate foehn penetration at very high resolution, we defined 78 vertical levels with the first mass level at 10 m a.g.l., and the model top at 22 km. To ensure a good vertical resolution within the boundary layer and to reduce computational costs by limiting the total number of vertical levels, the vertical grid spacing increases with altitude to 400 m at the model top. The time-step was decreased from 30 s in D1 to 3 s in D3.

Similar to GRAMM-SCI, land-use information comes from the CLC18 dataset. The land-use category in WRF is, however, defined as the dominant CLC18 class in the respective grid cell in contrast to GRAMM-SCI, which does not define a representative land-use class at all, but rather uses the median land-use parameters. The land-use classes and parameters in WRF and GRAMM-SCI can thus differ even though they both use the same CLC18 dataset.

Following Schicker et al. (2016), we re-classified the CLC18 dataset (44 categories) into the USGS land-use classes (33 categories) based on Pineda et al. (2004) to be able to use the already existing USGS parameter table. ERA5 reanalysis data were used for initial and boundary conditions with a temporal resolution of 1 h, a spatial resolution of 0.25$^{\circ }$, and 137 levels from the ground till 79 km a.g.l.

After testing different planetary boundary layer (PBL) schemes, we selected the Mellor-Yamada Nakanishi and Niino Level 2.5 (MYNN2, Nakanishi 2001; Nakanishi and Niino 2004) parameterization. This model is based on the Mellor-Yamada-Janjic turbulence kinetic energy (TKE) closure (Mellor and Yamada 1974). The widely used Noah land-surface model (LSM, Chen and Dudhia 2001) was used in the “dominant category” mode, which means that, if the land-use dataset has a higher resolution than the model domain, the land-use parameters are determined according to the land-use class covering the largest fraction of the model grid cell. Surface-atmosphere exchange is computed using the revised MM5 surface-layer scheme (Jiménez et al. 2012). The same surface-layer formulation is also used during post-processing to extrapolate wind and temperature from the first model level to the height of the measurements for model evaluation in a post-processing step. Modeled temperature is additionally corrected for elevation differences between the model topography and the real terrain using a constant lapse rate of 6.5 K km$^{-1}$ for both models.

The PBL parameterization, LSM, and surface-layer parameterization are called at every integration time-step. The radiative transfer is calculated every 5 min by the Rapid Radiative Transfer Model for longwave radiation (Mlawer et al. 1997) and following Iacono et al. (2008) for shortwave radiation. Sub-grid scale cumulus clouds—if there are any—are modeled by the updated Kain-Fritsch scheme (Kain 2004), which is also called every 5 min. Micro-physical sub-grid scale processes are represented by the Eta scheme (Rogers et al. 2001). For synoptically undisturbed and fair-weather conditions, clouds play a negligible role, so that the microphysics and cumulus parameterizations have almost no effect in our simulations and thus only simple, widely used schemes are activated to represent these sub-grid scale processes.

## Study Area and Numerical Experiments

### Measurement Sites

**Fig. 2:**
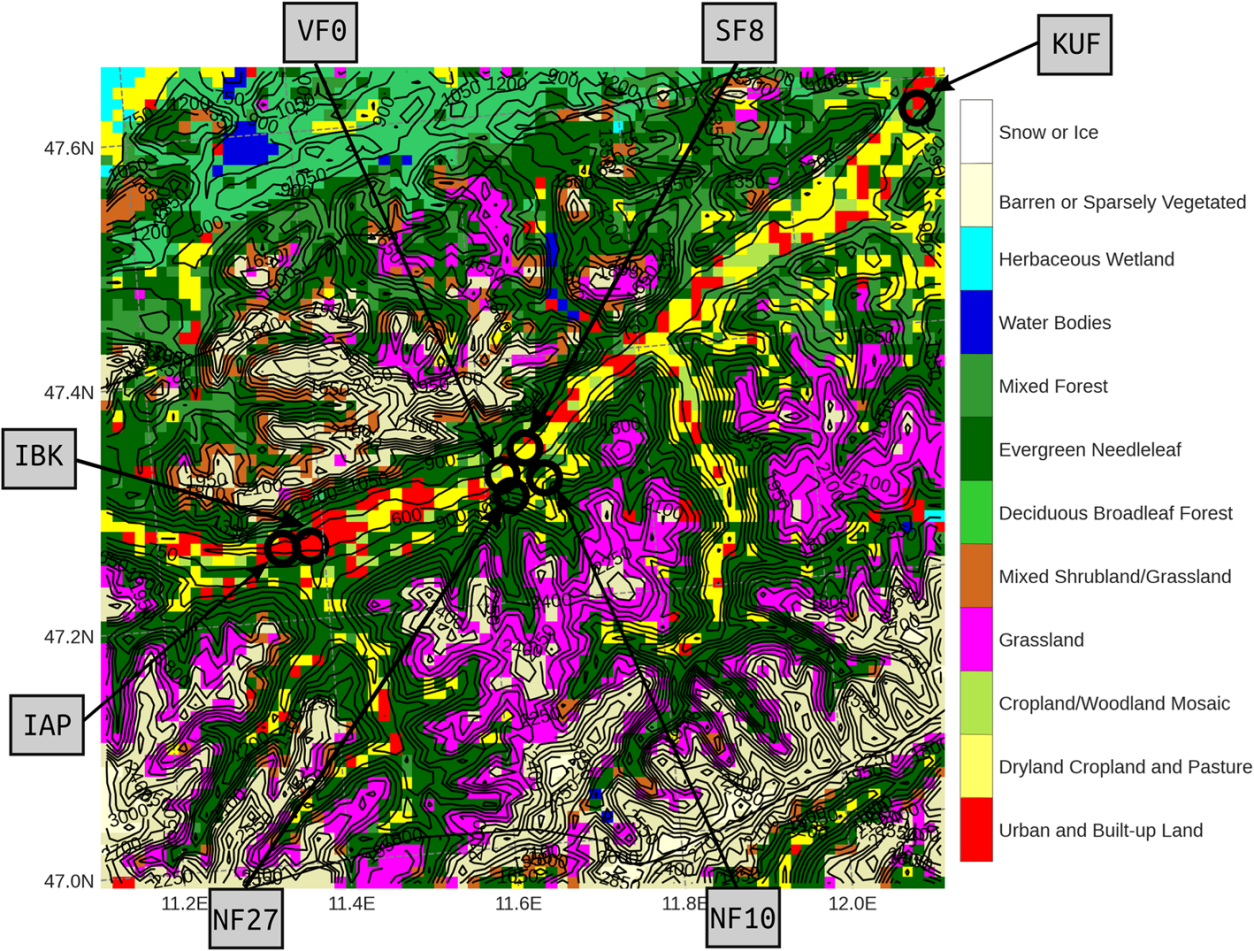
Dominant re-classified CLC18 land-use class used in the 1-km WRF grid and elevation contour lines (every 150 m) over the area of interest. Measurement stations used for model evaluation are represented with black circles

The model results are evaluated against observations from the Inn Valley in the western part of Austria, specifically an approximately 20-km long section of the valley going east from Innsbruck (Fig. 2). The Inn Valley is oriented approximately south-west to northeast and it opens to the German Alpine Foreland to the northeast of our area of interest. The valley floor has a width of about 2 km and the mountains rise to about 2000 m above the valley floor. Radiosoundings are launched every night at 0300 UTC at the Innsbruck airport (IAP), which are used to evaluate the models’ performance in reproducing the night-time vertical structure of the valley atmosphere. Additional profiles of wind speed and direction are available from a Doppler wind lidar (HALO Photonics, Lumibird, Lannion, France) operated at the roof of a university building in Innsbruck (IBK), approximately 35 m a.g.l. To calculate the horizontal pressure gradient along the valley, measurements are used from two automatic weather stations of the Austrian weather service GeoSphere Austria. One station is located at IAP and the second one in Kufstein at the valley exit (KUF in Fig. 2 ).

Modeled surface-energy budget components are evaluated against measurements from four eddy-covariance stations located approximately 20 km east of Innsbruck (Fig. 2). The stations are part of the i-Box (Innsbruck Box) project, which was designed to study boundary-layer processes in mountainous terrain (Rotach et al. 2017). Locations for the measurement sites were selected to represent different slope orientations, slope angles, and land-use characteristics (Table 1). The valley-floor site (VF0) on the almost flat valley floor is mainly surrounded by agricultural fields. Two sites are located on the north-facing sidewall on slopes with slope angles of about 10$^{\circ }$ (NF10) and 27$^{\circ }$ (NF27). The vegetation in the immediate vicinity of both sites is mainly grassland, but the area both up and down the slope of NF27 is also strongly forested. The fourth site, SF8, is located at the foot of the south-facing sidewall, close to a small embankment between a concrete helicopter landing zone and agricultural land. Orthophotos of the sites and their immediate surroundings together with the flux footprint areas are shown in Lehner et al. (2021, their Fig. 2).

**Table 1:** Characteristics of the i-Box stations used for model evaluation: elevation, land-use, slope angle $\alpha$ and orientation $\beta$, and measurement levels for sonic anemometers and gas analyzers $z_{son}$ and for temperature sensors $z_{T}$

Station	*Z* (m a.s.l.)	Land use	$\alpha$/$\beta$ ($^{\circ }$)	$z_{son}$ (m a.g.l.)	$z_{T}$ (m a.g.l.)
VF0	545	Mixed agricult	0.5/–	4.0, 8.7$^a$, 16.9	2.0
NF27	1009	Alpine meadow	25/1	6.8	6.8
NF10	930	Alpine meadow	11/345	5.7	5.3
SF8	575	Agricult./asphalt	9/147	11.2	11.2

$^\textrm{a}$No gas analyzer at this level

All four i-Box stations are instrumented with CSAT3 sonic anemometers (Campbell Scientific Ltd., Logan, UT, USA) and fast-response hygrometers (KH20 or EC150, Campbell Scientific Ltd.) at one or more vertical levels (Rotach et al. 2017; Lehner et al. 2021). Turbulent fluxes are calculated using the EdiRe Data Software as described in Lehner et al. (2021), following recommendations by Stiperski and Rotach (2016). Air temperature and humidity measurements for flux corrections and model evaluation come from PT100 temperature and HT-1 humidity sensors (HC2A-S, Rotronic, Bassersdorf, Switzerland). VF0, NF10, and NF27 are full surface-energy balance stations, with four-component radiation and soil measurements. Radiation is measured with CNR4 net radiometers (Kipp & Zonen, Delft, The Netherlands) at NF10 and NF27 and with a combination of CMP21 pyranometers and CGR4 pyrgeometers (Kipp & Zonen) at VF0. Soil heat flux plates (HFP01, Hukseflux, Delft, The Netherlands) are installed at a depth of 10 cm, with soil temperature and moisture probes (TRIME-PICO, IMKO, Ettlingen, Germany) between the heat flux plates and the surface to determine the storage term needed to calculate the surface ground heat flux (e.g., Foken 2017).

Figure 2 shows the land-use class used in the 1-km WRF domain. Because of the relatively coarse resolution of the models compared to the CLC18 dataset, multiple different land-use classes can be found within each grid cell and the land-use parameters used in WRF and GRAMM-SCI can differ, with WRF using the parameters of the dominant class and GRAMM-SCI the median (Sect. 2). Some of the key parameters at each of the i-Box sites are listed in Table 2 for both models. In WRF, all sites are classified as “dryland, cropland and pasture”, except for NF27, which is classified as “evergreen needleleaf forest”, while the station is actually located on grassland. In GRAMM-SCI, both NF27 and NF10 are dominated by coniferous forests, while the remaining sites are treated as pasture, which is in agreement with the conditions found at the station locations.

**Table 2:** Land-use characteristics at the grid cells representing the i-Box stations in WRF and GRAMM-SCI: percentage of forest cover, albedo *a*, emissivity $\epsilon$, roughness length $z_0$, and land-use. Parameters in GRAMM-SCI are based on median values over all land-use types within the grid cell and not on the dominant class indicated in the last column

Model	Station	Forest cover (%)	*a*	$\epsilon$	$z_0$	Land use
WRF	VF0	7	0.17	0.985	0.15	Dryland, cropland and pasture
	NF27	76	0.12	0.95	0.5	Evergreen Needleleaf Forest
	NF10	18	0.17	0.985	0.15	Dryland, cropland and pasture
	SF8	33	0.17	0.985	0.15	Dryland, cropland and pasture
GRAMM-SCI	VF0	0	0.19	0.92	0.1	Pasture
	NF27	79	0.12	0.66	1	Coniferous Forest
	NF10	57	0.12	0.73	1	Coniferous Forest
	SF8	22	0.19	0.92	0.1	Pasture

### Model Simulations

We selected a clear-sky and undisturbed summer day when thermally driven valley winds were observed. Lehner et al. (2019) have developed an objective selection technique based on 700-hPa geopotential-height gradients and measurements of incoming longwave radiation to identify valley-wind days in the Inn Valley. From this list, we selected 19 August 2018 because of the good data coverage at the measurement sites.

**Fig. 3:**
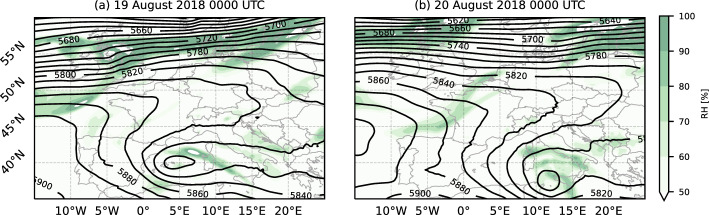
ERA5 geopotential height (black contour lines) and relative humidity (colour contours) at 500 hPa on **a** 19 August 0000 UTC and **b** 20 August 0000 UTC

The synoptic situation was characterized by an upper-level ridge over the Atlantic, extending over western France and Germany, and a low-pressure system over the Mediterranean Sea (Fig. 3). Geopotential-height gradients were weak over Austria and the region of interest, resulting in a weak synoptic flow during this period.

In addition to the reference GRAMM-SCI simulation (*GRAMM-base*), two sensitivity tests were performed to test the impact of (i) the initial soil-moisture fields and (ii) the new modifications of the longwave radiation and land-atmosphere exchange over forested grid cells described in Sect. 2.1. For *GRAMM-dry*, the initial ERA5 soil-moisture field was multiplied by a factor of 0.38. The factor 0.38 was determined to match the initial soil moisture in the model to the observed value at VF0. For *GRAMM-forest*, the new modifications of the land-surface scheme for forested grid cells were turned off. Differences between *GRAMM-base* and *GRAMM-forest* are expected in particular at the two north-facing slope sites NF27 and NF10, where 79% and 57% of the respective grid cell are covered by forest in GRAMM-SCI, but potentially also in the overall valley atmosphere because of the large fraction of the valley being covered by forest in CLC18 (Fig. 2).

The performance of the three GRAMM-SCI simulations is compared to that of a single WRF run. Both WRF and GRAMM-SCI simulations are started on 18 August 2018 at 1200 UTC, with the first 12 h considered as spin-up time. The initialization time was selected after performing a sensitivity study with WRF, which revealed better agreement between observations and model output when the model was initialized during convective daytime conditions.

## Results

**Fig. 4:**
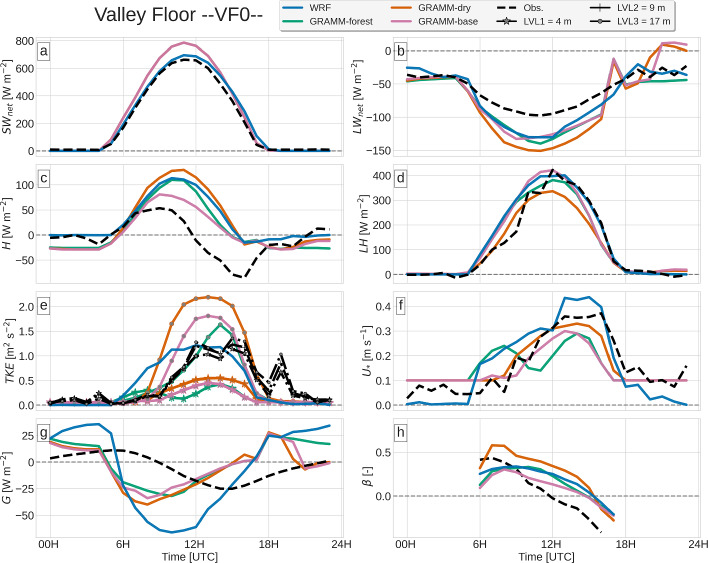
Observed and modeled time series of **a** net shortwave and **b** net longwave radiation, **c** sensible and **d** latent heat fluxes, **e** turbulent kinetic energy, **f** friction velocity, **g** ground heat flux, and **h** Bowen ratio. Observations of turbulence quantities are from 4 m a.g.l., except for TKE, which is shown for all three measurement levels

**Fig. 5:**
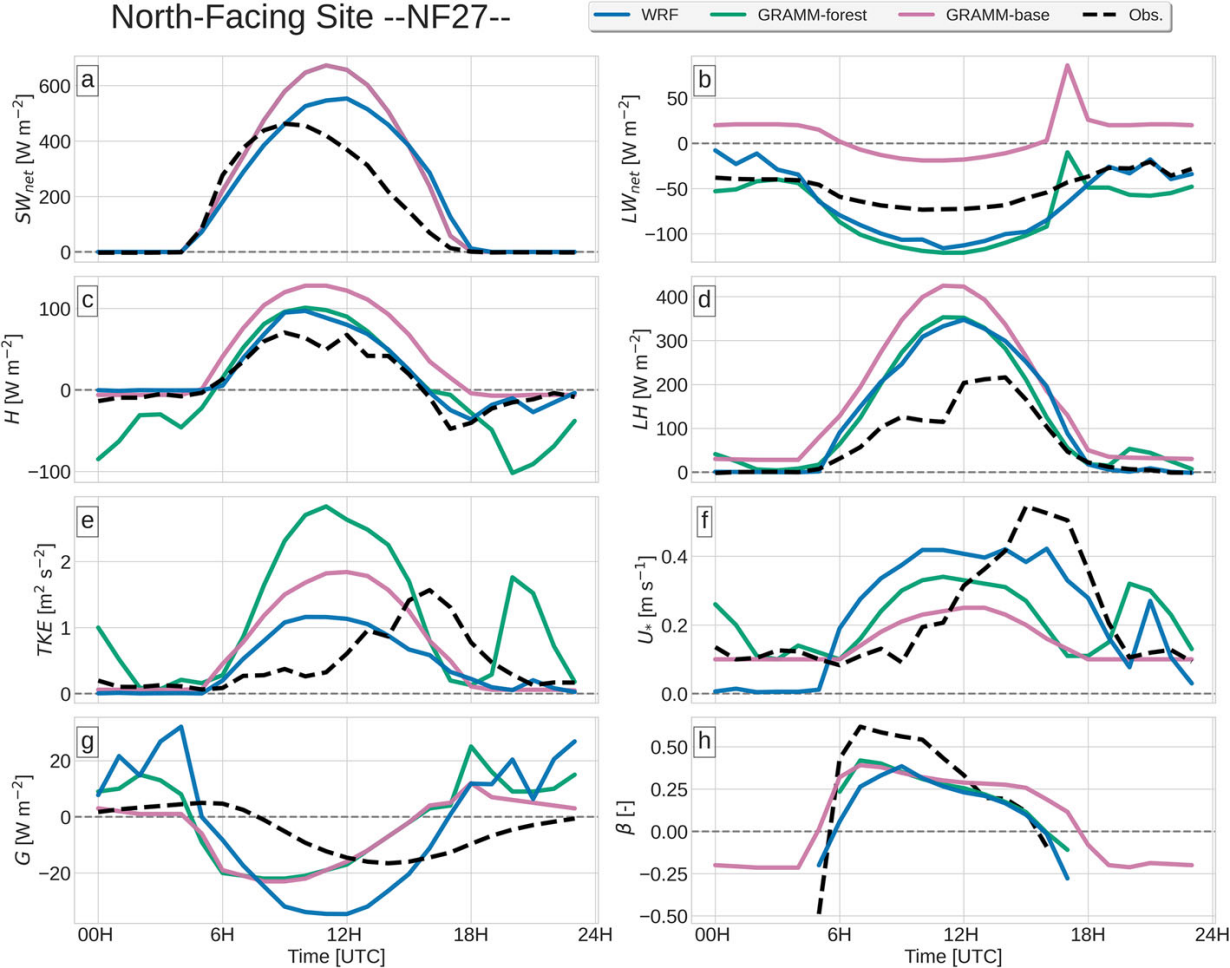
Same as Fig. 4, but for NF27

**Fig. 6:**
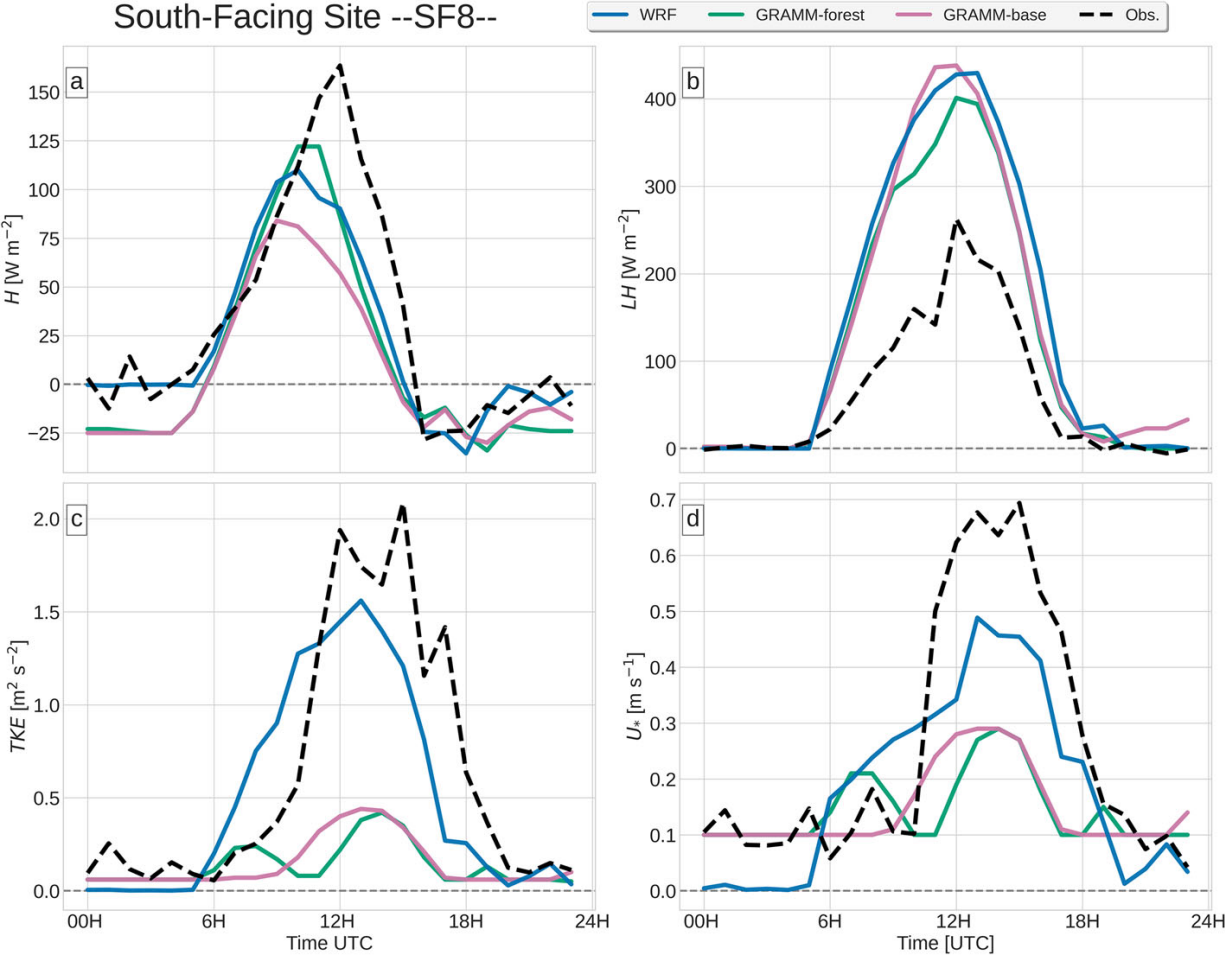
Observed and modeled time series of **a** sensible and **b** latent heat fluxes, **c** turbulence kinetic energy, and **d** friction velocity

In Sects. 4.1–4.4, the modeled Surface-Energy Budget (SEB) components and the turbulence kinetic energy are compared to the observations at VF0, NF27 and SF8 (Figs. 4, 5, 6). The models are then evaluated on their ability to reproduce near-surface and upper-air wind and temperature measurements in Secs. 4.5 and 4.6.

While measurements are made at three vertical levels at VF0 (Table 1), the comparison is mostly restricted to the lowest level at 4.0 m a.g.l., which is most representative of the surface layer. At SF8, no radiation and ground-heat flux measurements are made, so that the model evaluation is restricted to the turbulent heat fluxes. *GRAMM-dry* is only shown for VF0, for which the surface moisture was calibrated.

### Radiative Fluxes

Net shortwave radiation $SW_{net}$ at the almost flat valley floor is captured well by WRF, with both magnitude and timing matching the observations (Fig. 4a). The magnitude in GRAMM-SCI, on the other hand, is overestimated by almost 100 W m$^{-2}$. At the north-facing site NF27, both models fail to reproduce the observed diurnal cycle of $SW_{net}$, which is not symmetric with respect to noon, but shows a strong increase in intensity in the morning reaching its peak of 450 W m$^{-2}$ at 1000 UTC (Fig. 5a). The local slope at NF27 is oriented to the north, with a slope orientation of approximately 360$^{\circ }$. The shift in the observed maximum may be related to a slight deviation of the sensor orientation from north. The orientation of the WRF model terrain is close to the real topography, with an orientation angle of 355$^{\circ }$, but the modeled $SW_{net}$ reaches its maximum close to noon as expected for a north-facing slope. Similar to WRF, the peak in GRAMM-SCI is shifted towards noon. Using a higher horizontal resolution of 200 m does, however, result in a closer agreement with the observations, likely due to the model capturing small-scale terrain variations in the vicinity of the station (not shown). These discrepancies between the modeled and observed shortwave radiation occur only at NF27 and are not observed at NF10 (not shown), for which the results are similar to VF0 with GRAMM-SCI having an offset of 2 h, but reproducing the magnitude and WRF capturing the diurnal cycle well only with a slightly later decrease at noon. Differences between model and observations may also be partly explained by the fact that albedo changes during the day (Lehner et al. 2021), which is not taken into account in the models.

Both models overestimate net longwave radiation $LW_{net}$ at VF0 during the daytime (Fig. 4b). While $LW_{in}$ is close to the observed values in WRF, $LW_{out}$ is strongly overestimated by the model (not shown), suggesting that the model bias is a result of incorrect surface temperatures. GRAMM-SCI does not output the upward and downward components separately. To get some indication of whether GRAMM-SCI equally overestimates $LW_{out}$, we calculated $LW_{out}=443$ W m$^{-2}$ from the surface temperature at the time of the minimum in $LW_{net}$. The observed value at this time is 478 W m$^{-2}$ and the difference to the modeled value is in the range of uncertainty for the emissivity. $LW_{out}$ is thus somewhat underestimated by the model, which means that $LW_{in}$ must be equally underestimated, but with a larger bias to explain $LW_{net}$.

At NF27, *GRAMM-base* strongly underestimates the daytime magnitude of $LW_{net}$ and the offset is equally high during nighttime, with positive $LW_{net}$ in the model in contrast to the observed negative values. In GRAMM-SCI, the grid cell closest to NF27 is largely covered by forest, so that the model’s surface emissivity is not representative of the grassland at the station, in particular the modified emissivity to model the below-canopy longwave enhancement in forests. *GRAMM-forest*, for which the emissivity is not reduced artificially over forested areas, thus reproduces the observations much better, similar to WRF. It has to be pointed out, however, that the better agreement results from the fact that the model’s vegetation does not match the real vegetation at the site because of the coarse grid spacing, which does not capture small-scale changes in vegetation.

### Turbulent Heat Fluxes

In the Inn Valley, the latent heat flux *LH* typically exceeds the sensible heat flux *H* (Lehner et al. 2021), which can also be observed on 19 August 2018 (Figs. 4, 5, 6). Both models capture this behaviour in general, but the details in the partitioning of the available energy into sensible and latent heat fluxes differ from site to site and also among the simulations. WRF closely reproduces the observed *H* at NF27, but strongly overestimates *LH* during daytime (Fig. 5). This is also reflected in an underestimation of the Bowen ratio $\beta$ = $\frac{H}{LE}$, in particular in the morning. The partitioning of the available energy is thus biased towards *LH* at this site. At SF8, *LH* is equally overestimated and *H* is even slightly underestimated (Fig. 6). The same is also true for *GRAMM-base*, which overestimates *LH* at NF27 and SF8 similar to WRF, but underestimates *H* at SF8, while producing slightly higher values of *H* than observed at NF27. The large discrepancies at SF8 may be partly related to the location of the station close to a large concrete surface, which causes the highest $\beta$ among all i-Box sites (Lehner et al. 2021), whereas the site is characterized by cropland and pasture and by pasture in WRF and GRAMM-SCI, respectively. *GRAMM-forest* produces a large increase in *H* during daytime compared to *GRAMM-base* and thus a better agreement with the observations, similar to WRF, even though the forest cover is only 22% at this grid cell. Daytime *LH* is equally lower in *GRAMM-forest*, demonstrating a difference in the partitioning of the turbulent fluxes between the two simulations.

Interestingly, both models perform better in modeling the latent heat flux than the sensible heat flux at VF0 (Fig. 4), in contrast to NF27 and SF8. Similar to SF8, WRF and *GRAMM-forest* produce a larger *H* than *GRAMM-base*, with the magnitude in *GRAMM-base* closest to the observed daytime maximum and WRF and *GRAMM-forest* strongly overestimating observed values. Both at VF0 and SF8, the higher Bowen ratio in *GRAMM-forest* than in *GRAMM-base* around noon is accompanied by a lower friction velocity. This large difference between *GRAMM-base* and *GRAMM-forest* comes as a surprise since the forest cover at VF0 is zero in GRAMM-SCI. The results thus suggest that the influence of the changes in longwave radiation and land-atmosphere exchange over forested grid cells extends beyond the immediately affected grid cells.

In contrast to NF27, the observed sensible heat flux at VF0 peaks already before the time of the maximum in $SW_{net}$ and changes sign shortly afterwards to become negative around the time of maximum irradiation. This is a characteristic at VF0, where the maximum *H* is typically reached at approximately the transition time from nighttime down-valley to daytime up-valley winds (Lehner et al. 2021), which can also be observed on 19 August 2018 (compare Fig. 4c to Fig. 8a). While all four simulations produce a slightly earlier peak in *H* than in *LE*, the modeled peak occurs overall later than in the observations, with *GRAMM-base* closest to the observed timing. Together with the general overestimation of *H*, this results in too high Bowen ratios in the afternoon compared to the observations. The modeled Bowen ratio actually remains relatively constant throughout the day at both VF0 and NF27, whereas the observed $\beta$ decreases with time, indicative of a temporal shift in the maxima of *H* and *LH*, which are not captured correctly by the models.

While WRF can reproduce the close-to-zero turbulent fluxes during nighttime, GRAMM-SCI generally overestimates the magnitude of either *H* or *LH* by up to about 25 W m$^{-2}$. At VF0 and SF8, all GRAMM-SCI simulations produce large negative *H* during the night, while *GRAMM-base* overestimates *LH* at NF27. *GRAMM-forest*, which does not include the modified drag law over forested areas, overestimates nighttime magnitudes of *H* by up to 100 W m$^{-2}$ and *LH* by up to 50 W m$^{-2}$, highlighting the benefit of the new modifications implemented in GRAMM-SCI. The applicability of a direct comparison between modeled values of turbulent fluxes and observations at NF27 is, however, somewhat limited by the fact that the vegetation in the nearest grid cell is largely forest in both models in contrast to the grassland covering most of the flux footprint area at the station.

### Surface Energy Budget

Another challenge when evaluating modeled sensible and latent heat fluxes with measurements is that many observational studies have revealed an under-closure of the surface-energy budget (Wilson et al. 2002; Grachev et al. 2020; Mauder et al. 2020; Lehner et al. 2021), leading to a large residual of the surface-energy budget:

5 $$\begin{aligned} Res = R_{net} - (H + LH + G), \end{aligned}$$

where $R_{net}=SW_{net}+LW_{net}$ is the net radiation and *G* is the ground heat flux. Lehner et al. (2021) have shown that the residual can actually reach magnitudes similar to the sensible heat flux in the Inn Valley. This under-closure is generally assumed to be the result of advection by quasi-stationary circulation systems and large eddies, which result from surface heterogeneities and are not captured by traditional eddy-covariance systems (Mauder et al. 2020), as well as of within-canopy storage.

Numerical models, like WRF and GRAMM-SCI, on the other hand, do not take advection or storage terms into account and the surface-energy budget is closed by definition. A direct comparison of modeled and observed turbulent fluxes can thus not be expected to yield a perfect agreement if the observed surface-energy budget is not closed. In particular during daytime, when fluxes are overall larger than during nighttime, this may lead to large absolute differences between the model and the observations. To better judge the model performance, we want to determine whether the general overestimation of the turbulent fluxes by the two models matches the observed residual.

Assuming that there is no difference between the observed and the modeled $R_{net}$, we define $\phi _{mod}$ as the sum of all deviations between modeled and observed fluxes:

6 $$\begin{aligned} \phi _{mod} = (H_{mod,t}-H_{obs,t})+ (LH_{mod,t}-LH_{obs,t})+(G_{mod,t}-G_{obs,t}), \end{aligned}$$

where the indices *mod* and *obs* indicate the modeled and observed fluxes at each model output time *t*. $\phi _{mod}$ can be integrated over the whole day:

7 $$\begin{aligned} \varPhi _{mod} = \int _{t=\text {0 h}}^{t=\text {24 h}} \phi _{(mod,t)} dt= \text {3600s}\sum ^{t=23}_{t=0}\phi _{(mod,t)}, \end{aligned}$$

to give the total daily difference between modeled and observed energy fluxes. While $\varPhi _{mod}$ provides information on whether the overestimation of modeled turbulent fluxes can be explained by the observed non-closure of the surface-energy balance, it does not provide validation of the modeled partitioning of the available energy into sensible and latent heat fluxes. This is analyzed using the Bowen ratio in Figs. 4, 5, 6.

**Fig. 7:**
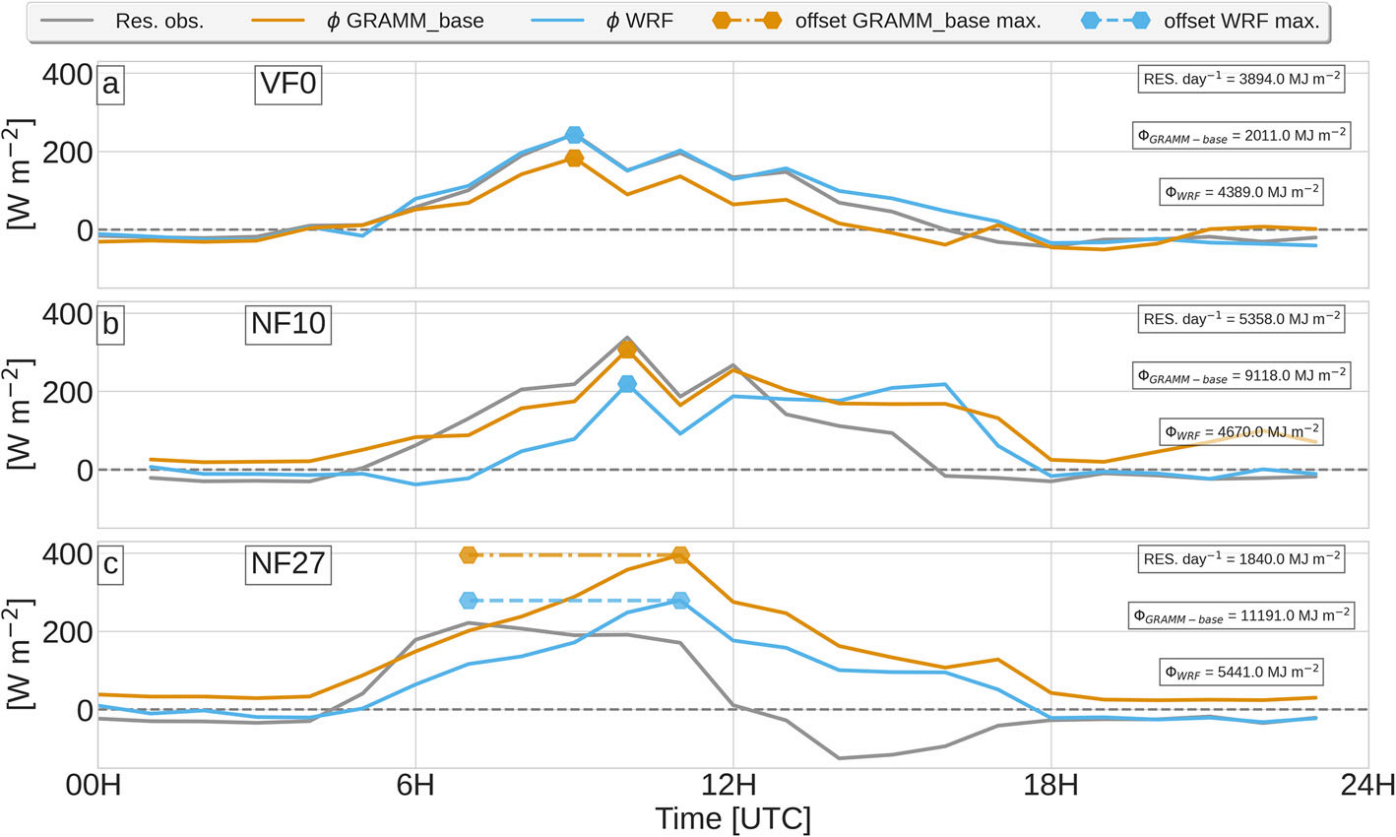
Residuals of the observed surface-energy budget (grey lines) and $\phi$ (Eq. 6) for *GRAMM-base* (orange lines) and WRF (blue lines) at **a** VF0, **b** NF10, and **c** NF27. The integrated model bias $\varPhi$ (Eq. 7) is indicated in the top right of each subfigure together with the daily sum of the observed residuals. Horizontal lines connect the location of the peak in the observed residual with the respective peak in $\phi _{mod}$

Figure 7 presents the residuals in the surface-energy budget together with $\phi$ for VF0, NF10, and NF27. The residual cannot be calculated for SF8 since radiation and ground heat flux are not measured at the site. The magnitude of the observed residual varies somewhat among the sites. At NF10, the largest value occurs at 1000 UTC with 375 W m$^{-2}$, followed by VF0 with a peak of 240 W m$^{-2}$ one hour earlier at 0900 UTC and, finally, by NF27 with a peak of 210 W m$^{-2}$ at 0700 UTC. At the valley floor, $\phi _{WRF}$ closely follows the observed residual (Fig. 7a). The time of the peak at 1000 UTC agrees with the observed peak and $\varPhi _{WRF}=4389$ MJ m$^{-2}$ is only slightly higher than the total residual of 3894 MJ m$^{-2}$. This means that the differences between the modeled and observed turbulent fluxes at VF0 can be explained by the observed residual, which is distributed among the other fluxes in the model. In *GRAMM-base*, the maximum of $\phi _{GRAMM}$ is also coinciding with the observed peak and the peak in $\varPhi _{WRF}$ but the daily total $\varPhi _{GRAMM}=2011$ MJ m$^{-2}$ is much lower than the daily sum of the residual. This comparison between the observed residual and $\phi _{mod}$ is based on the assumption that the model captures the net radiation, that is, the available energy, correctly. While, in particular $SW_{net}$ is captured well by WRF (Fig. 4a), in GRAMM-SCI the magnitude of $SW_{net}$ is about 150 Wm$^{-2}$ too large, which may explain part of the differences between $\phi _{GRAMM}$ and the observed residual. The fact that the agreement between *Res* and $\phi$ is better during nighttime corroborates that the daytime deviations may be related to differences in radiation.

At NF10, the timing of the maximum in both $\phi _{WRF}$ and $\phi _{GRAMM}$ agrees with the timing of the peak in the observed residual as well. The values of $\phi _{WRF}$ remain, however, high until the evening and the observed maximum residual is underestimated by almost 30%. While $\phi _{GRAMM}$ captures the magnitude of the observed peak, it overestimates the observed residual during nighttime, in particular during the second night. The daily totals, on the other hand, show that, while $\varPhi _{GRAMM}=9118$ MJ m$^{-2}$ is about 1.5 times as high as the daily total observed residual (5358 MJ m$^{-2}$), $\varPhi _{WRF}=4670$ MJ m$^{-2}$ is relatively close and only the timing is not captured correctly. Similar results are found at NF27, with both models having the same 4-h offset between the peak in the observed residual and the peak in $\phi _{mod}$. The daily total residual (1840 MJ m$^{-2}$) is, however, strongly overestimated by both models with $\varPhi _{GRAMM}=11191$ MJ m$^{-2}$ and $\varPhi _{WRF}=5441$ MJ m$^{-2}$. The observed residual actually changes sign at 1200 UTC to become negative, which is not recreated by either model. Part of the larger differences at the slope sites may be again related to the violation of the basic assumption that the model captures the net radiation correctly (Fig. 5). Another possible explanation can be found in the aforementioned tendency of the models to produce the peaks in *H* and *LH* around noon and in the ground heat flux *G*, whose diurnal cycle is badly captured by both models at NF27 (Fig. 5), and also VF0 (Fig. 4). While the observed *G* reaches its maximum in the afternoon, WRF produces the maximum around 1200 UTC, and GRAMM-SCI in the morning. At the moment, we can, however, offer no explanation for the discrepancies among the models. Lehner et al. (2021) have mentioned that the residual decreases in the afternoon because of large values in *LH* and because of the peak in *G*. The early modeled maximum in *G* thus reduces $\phi$ in the morning instead of the afternoon as observed.

During nighttime, the observed residual is generally negative, that is, the turbulent fluxes exceed the available energy, consistent with findings by Grachev et al. (2020). $\phi _{WRF}$ is equally negative, particularly during the second night, when it follows the observed residual closely. This means that the observed surface-energy imbalance can also partly explain the slightly underestimated turbulent nighttime fluxes in the model. In GRAMM-SCI, which generally overestimates turbulent fluxes even during nighttime, $\phi _{GRAMM}$ remains positive during nighttime, except for VF0.

### Turbulence Kinetic Energy and Friction Velocity

Momentum exchange at the surface is represented by $u_*$ in Figs. 4, 5, 6, which is generally weak in the morning before the onset of the up-valley flow (see Sect 4.5) and then peaks in the late afternoon. As described in Sect. 2.1, the roughness length in GRAMM-SCI cannot exceed 0.1 m to avoid unrealistically high turbulent fluxes. This also affects $u_*$ in regions with higher roughness length, for example, over the forested area at NF27, where $u_*$ remains very low throughout the day in *GRAMM-base*. In addition to the upper threshold for the roughness length, $u_*$ itself has also a lower threshold of 0.1 m s$^{-1}$ in GRAMM-SCI to avoid a decoupling of the surface under very stable conditions, which may explain the overestimation of either H or LH during nighttime. Performing a test simulation with a threshold value of 0.15 m $\textrm{s}^{-1}$ led to an even stronger overestimation. WRF, which captures the low nighttime turbulent fluxes better, actually underestimates $u_*$ with values close to 0 m s$^{-1}$ in contrast to the observed values of about 0.1 m s$^{-1}$.

TKE follows a similar diurnal cycle as $u_*$. While WRF captures the magnitude relatively well, with a maximum underestimation of about 0.5 m$^2$ s$^{-2}$ at SF8, it cannot reproduce the timing. Similar to $u_*$, TKE starts to increase already in the early morning around sunrise, whereas the measurements indicate an increase only later in the day together with the onset of the up-valley flow. WRF thus strongly overestimates TKE in the morning. The model does not produce an early increase in wind speed (see Sect. 4.5), so that the wind speed is still relatively low in the morning. Only buoyancy production contributes to the increase in TKE at this time, while shear production becomes relevant together with the increase in the along-valley wind later in the day (not shown). GRAMM-SCI, on the other hand, captures the diurnal cycle of TKE better, in particular at VF0 (Fig. 4g), but generally underestimates the magnitudes at the first model level at 10 m above ground level (i.e. in the surface layer), while magnitudes are slightly overestimated higher up. Hence, the model produces a strong increase of TKE with height, which is not observed. TKE at the first model level is not calculated using the prognostic equation, but is diagnosed from $u_*$ (Eq. 2). It seems thus that the surface-layer diagnostic equation in GRAMM-SCI has problems reproducing observed near-surface TKE.

### Surface Temperature and Wind

**Fig. 8:**
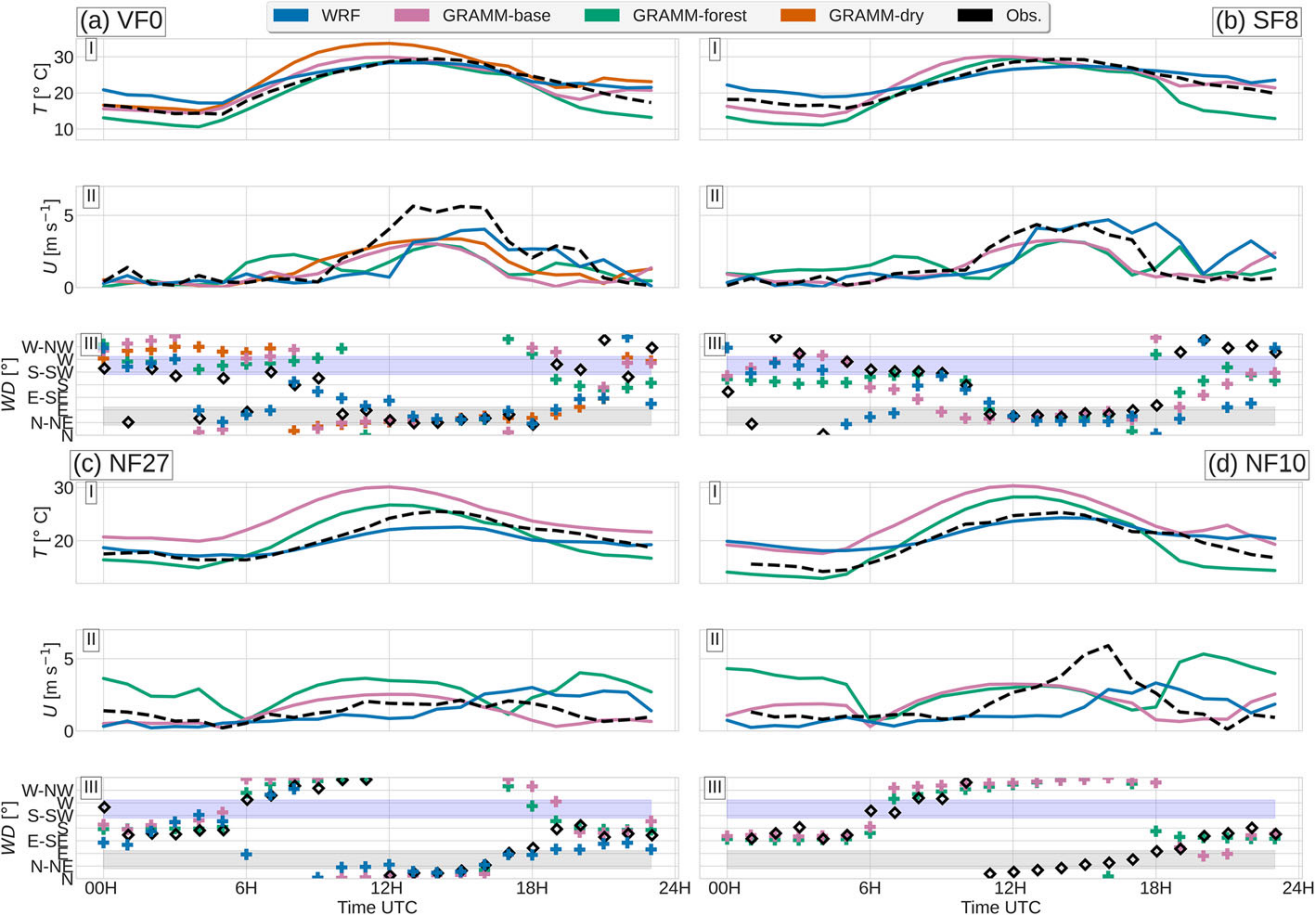
Time series of (I) temperature, (II) wind speed, and (III) wind direction at **a** VF0, **b** SF8, **c** NF10, and **d** NF27. The shaded areas in each subfigure (III) indicate the up-valley (grey) and down-valley (purple) direction

**Fig. 9:**
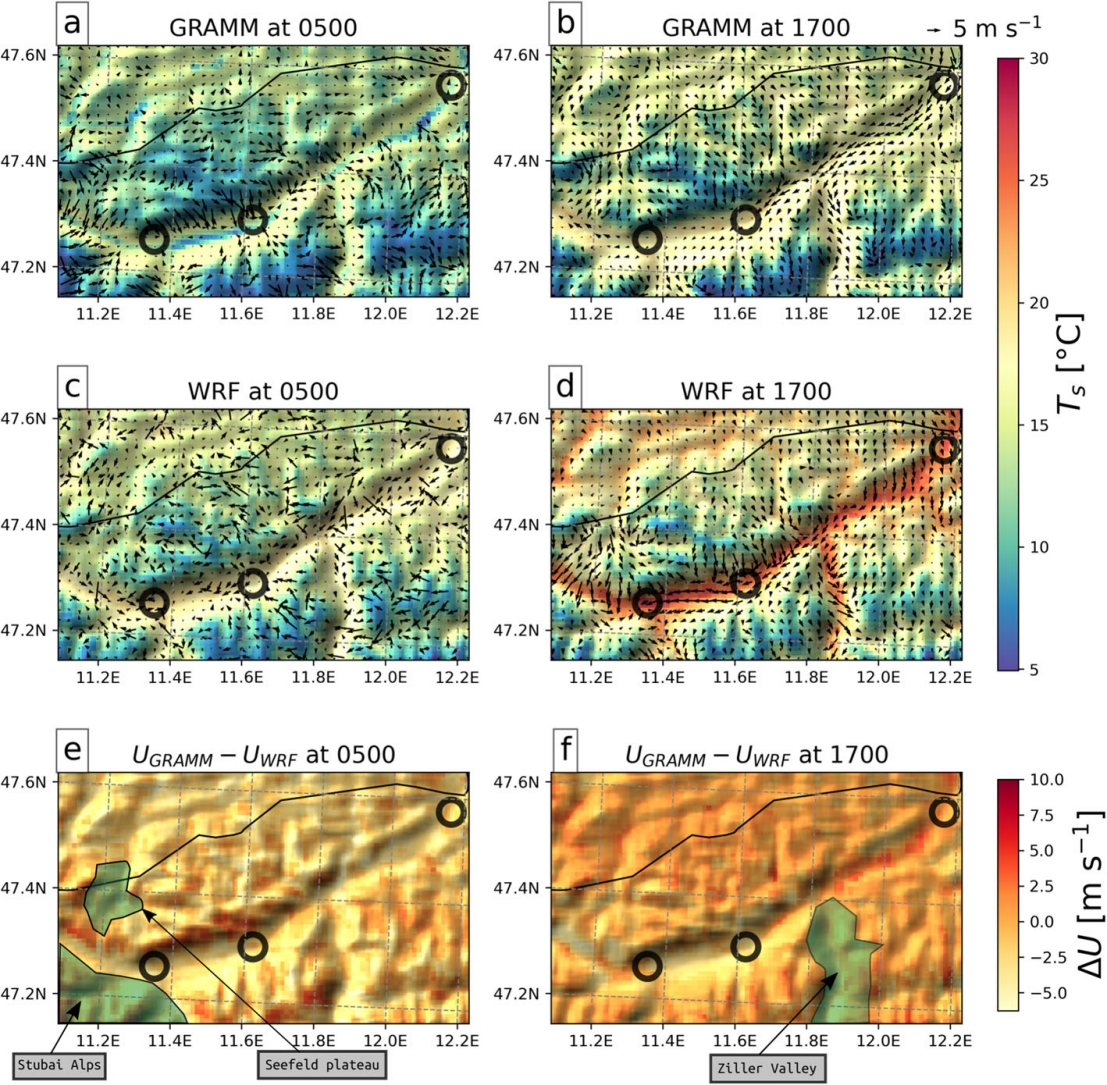
**a**–**b** *GRAMM-base* and **c**–**d** WRF wind speed and direction at 10 m a.g.l. (arrows) and 2-m temperature (colour contours) **a**,**c** during the down-valley period at 0500 UTC and **b**,**d** during the up-valley period at 1700 UTC. **e**–**f** Difference in wind speed between GRAMM-SCI and WRF ($U_{GRAMM}-U_{WRF}$) at **e** 0500 and **f** 1700 UTC. Black circles indicate the locations of IAP (leftmost), VF0 (middle), and KUF (rightmost)

In this section we focus on evaluating the models’ surface temperature and wind fields. Correct surface wind fields are crucial for air-pollutant transport modeling, and the modeled fields are tightly linked to the surface-energy budget discussed in the previous subsection. Time series of near-surface wind and temperature at the slope sites NF27, NF10, and SF8 and at the valley floor VF0 are shown in Fig. 8. The temperature has a similar diurnal cycle at all four sites, starting to rise around 0500 UTC to reach its maximum around 1400 UTC. WRF mostly underestimates the diurnal cycle slightly, overestimating nighttime and underestimating maximum daytime temperatures. *GRAMM-base* reproduces the diurnal cycle better at VF0 and SF8 than at NF10 and NF27, with a slight tendency to underestimate temperatures in the morning at SF8. On the north-facing sidewall, temperatures remain too high throughout the day. Horizontal cross-sections of the surface temperature in Fig. 9 show, however, that temperatures are relatively homogeneous along the valley floor in WRF both in the morning and in the evening, whereas temperatures are spatially more heterogeneous in *GRAMM-base*, with patches of colder and warmer air. The temporal evolution at individual grid cells depends thus on the exact location. *GRAMM-forest* reproduces the observed temperature time series at NF10 and NF27 best, except for the second night. Since net longwave radiation is captured better by *GRAMM-forest* than *GRAMM-base* (Fig. 5), this may explain the closer agreement in temperature. At VF0 and SF8, *GRAMM-forest* causes stronger cooling than *GRAMM-base*, likely as a result of a slightly larger negative sensible heat flux during nighttime (Figs. 4 and 5). Similarly, the higher daytime sensible heat flux in *GRAMM-dry* compared to the other simulations leads to the highest temperatures during daytime at VF0, overestimating the observed values, but resulting in a better agreement with observations than *GRAMM-base* during the late afternoon transition period.

**Fig. 10:**
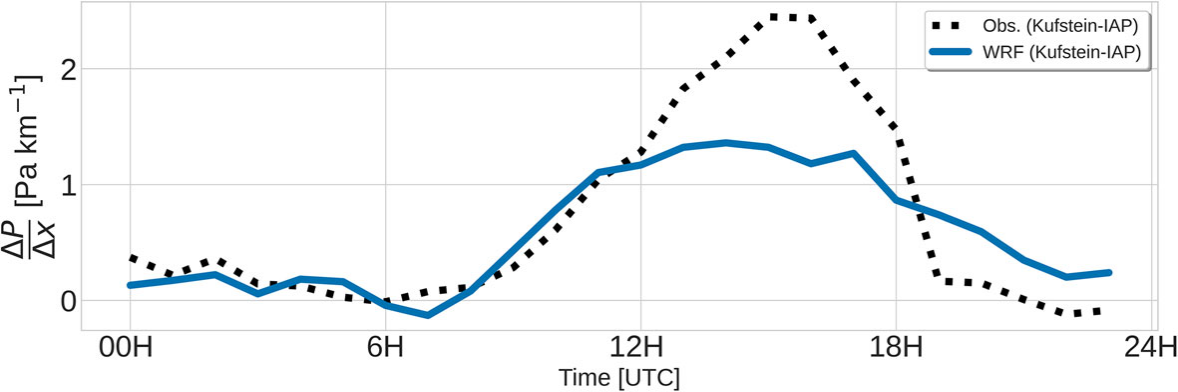
Daily cycle of the observed and modeled horizontal pressure gradient between Kufstein and Innsbruck airport. Pressure at Innsbruck was hydrostatically reduced to the altitude of Kufstein before calculating the horizontal gradient

Observed wind speeds at the valley floor are very weak during the night, without a pronounced down-valley flow, but with wind directions rather switching between down-valley and up-valley (Fig. 8a). The flow transitions to a steady up-valley direction around 1000 UTC, together with an increase in wind speed, which reaches its maximum in the afternoon hours. Both models capture the low wind speeds during nighttime, with *GRAMM-base* producing a more northerly flow component at VF0, while WRF reproduces the predominance of the along-valley direction and at least one switch between up-valley and down-valley directions. While the onset of the up-valley flow is captured well in *GRAMM-base* and *GRAMM-dry*, it is delayed by about 2 h in WRF, as well as in *GRAMM-forest*. Daniels et al. (2006) analyzed model simulations for a case study in the Riviera Valley, Switzerland, and found that initial soil moisture fields have a large impact on the model’s skill in reproducing the valley-wind transition and the wind speed. While the reduction in soil moisture by a factor of 0.38 does not lead to a pronounced shift in the onset of the up-valley flow in our study, the associated increase in sensible heat flux and the thus higher temperatures result in a faster increase in wind speeds in the morning. All four model simulations underestimate the afternoon wind-speed maximum by about 2 m s$^{-1}$. This is related to an underestimation of the horizontal pressure gradient between Kufstein at the entrance of the Inn Valley and Innsbruck airport in the middle of the valley (Fig. 10). During the up-valley period between 1200 and 1800 UTC, the gradient in WRF is about 1 Pa km$^{-1}$ lower than in the observations. Interestingly, all simulations capture the wind speed at SF8 better, which is equally dominated by the up-valley flow during the afternoon (Fig. 8b). While surface up-valley winds in the Inn Valley occur mainly in the valley section between Innsbruck and the Ziller Valley in WRF, as well as at the entrance of the valley, near-surface up-valley winds are strongest east of VF0 in GRAMM-SCI (Fig. 9). Up-valley winds form also in the tributary valleys to the south, such as the Ziller Valley and the Wipp Valley south of Innsbruck. This means that both models can at least qualitatively capture smaller valley-wind systems with the 1-km horizontal resolution. Without further observational data for model comparison, we can, however, not judge whether the models can also reproduce the strength of the valley winds, which Schmidli et al. (2018) have shown to be underestimated in small valleys with insufficient horizontal resolution.

At NF10 and NF27, slope winds dominate the wind field during most of the day (Figs. 8c, d). Nighttime southerly downslope winds are replaced by northerly upslope flows starting around 0600 UTC, with the onset of surface heating. In the afternoon, the flow becomes more strongly influenced by the easterly up-valley flow, in particular at NF10. This influence of the up-valley flow is also reflected in the increasing wind speed during the afternoon, while wind speeds remain weak at NF27. All three simulations capture the changes in wind direction throughout the day well at both sites. *GRAMM-forest* strongly overestimates downslope winds during the nighttime. The modifications to the surface-layer scheme in *GRAMM-base* result in a drop in wind speed at NF27 and thus a better representation of the observations, which highlights the benefit of these modifications for forested areas. At NF10, the reduction in nighttime wind speed is, however, somewhat smaller than at NF27 because of the lower percentage of forest cover and the correspondingly lower drag force at NF10 (Table 2). WRF produces also strong northerly winds into the Inn Valley in the evening, for example, along the valley sidewall north of VF0 and flows off the Seefeld Plateau west of Innsbruck (Fig. 9d). Since these northerly flows start to develop around 1000 UTC, they may be related to a larger-scale plain-mountain circulation between the northern German foreland and the Alpine range.

GRAMM-SCI produces overall higher wind speeds near the mountain tops than WRF, which can be seen well in the difference between the two models in Fig. 9e, f. In the early morning, the model simulates strong katabatic winds from the peaks to the north and south of the Inn Valley and in the evening, equally strong upslope flows mainly from a northerly direction. This is related to an overestimation of the upper-level flow in GRAMM-SCI, which will be discussed further in the next subsection. WRF, on the other hand, produces some relatively strong up-valley winds in the early morning, specifically just west of VF0 and east of the entrance to the Ziller Valley (Fig. 9c), which cannot be verified or explained at the moment.

### Vertical Wind and Temperature Profiles

**Fig. 11:**
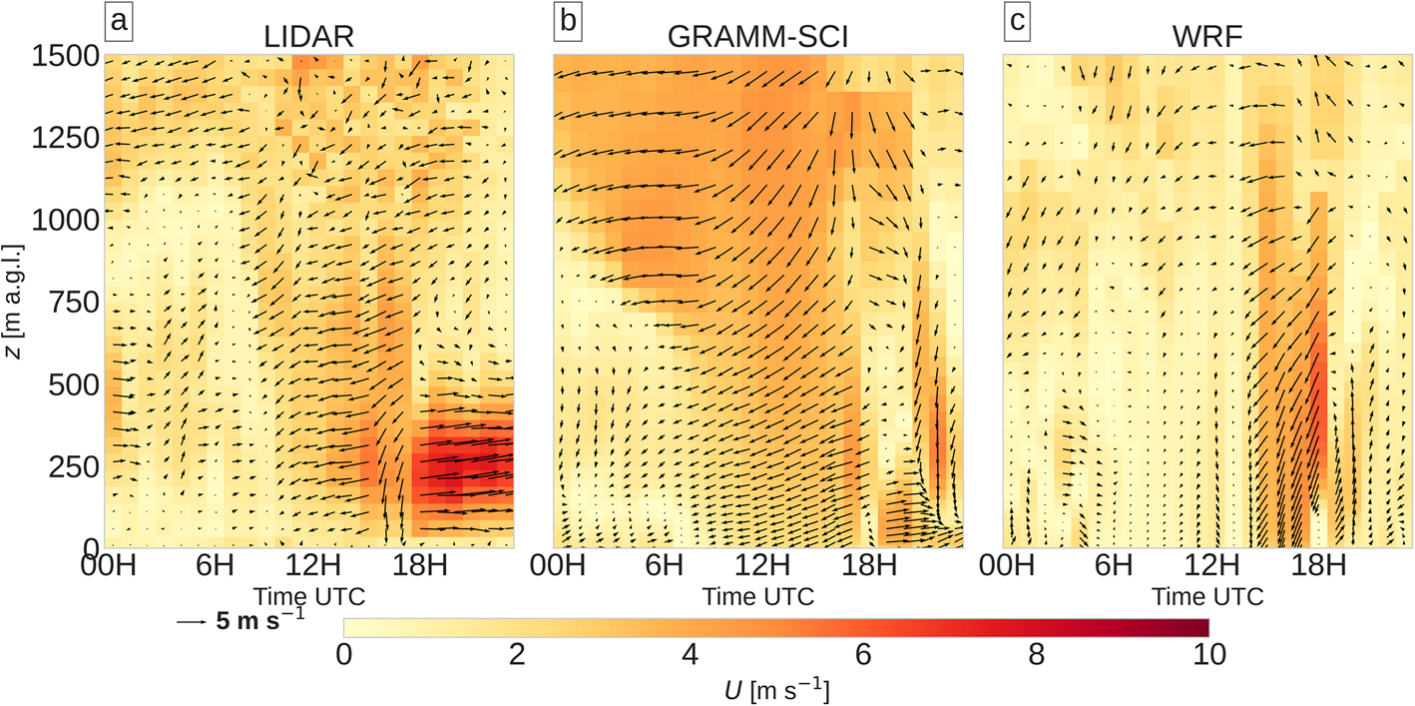
Wind speed (colour contours) and wind arrows at Innsbruck university from **a** lidar measurements, **b** *GRAMM-base*, and **c** WRF. Note that the height in **a** refers to above the location of the lidar, which is deployed on a rooftop 35 m a.g.l

**Fig. 12:**
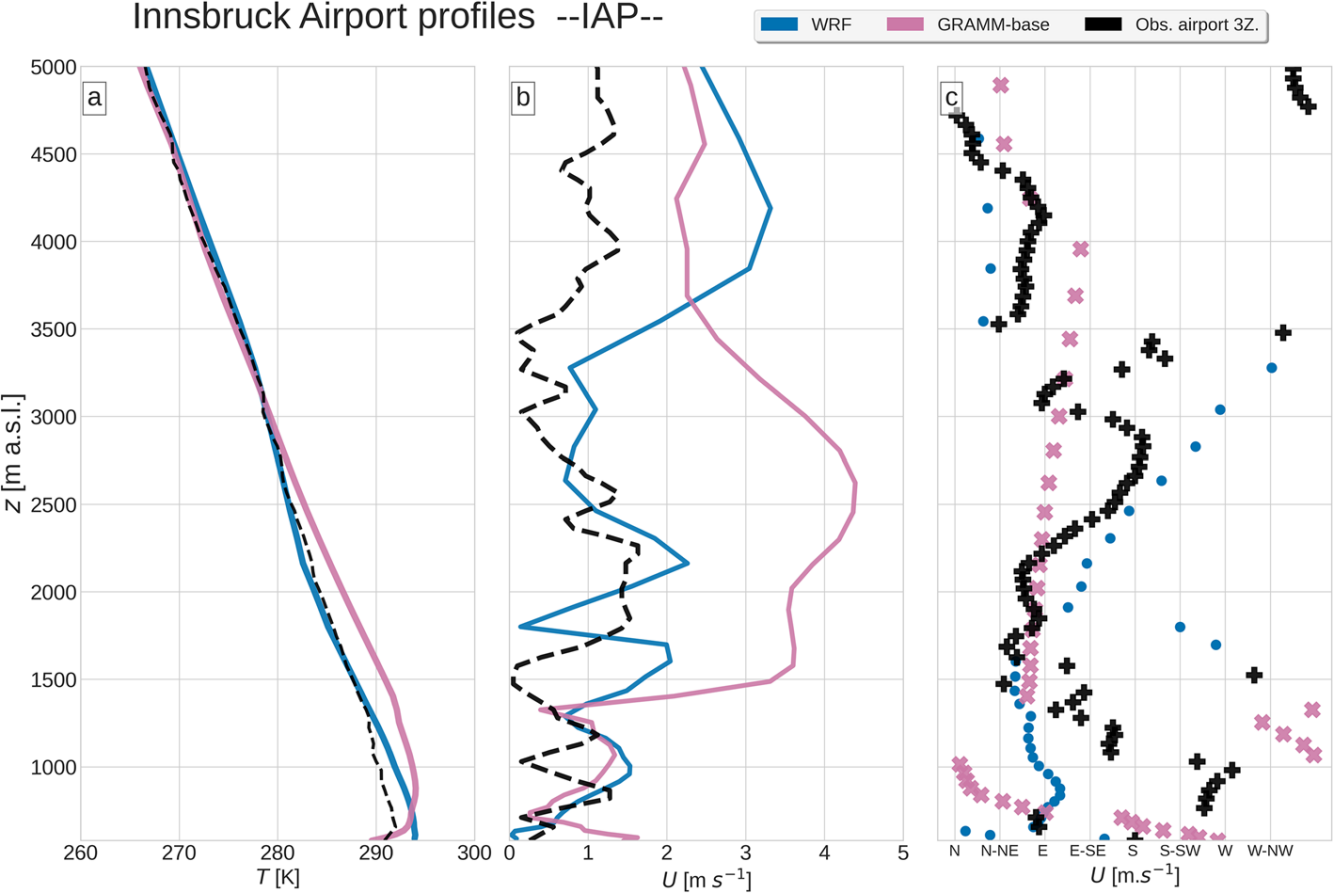
Vertical profiles from radiosondes and models of **a** temperature, **b** wind speed, and **c** wind direction at Innsbruck airport on 19 August 2018 at 0300 UTC

Figure 11 shows time-height cross-sections of the wind speed at IBK from lidar measurements, *GRAMM-base*, and WRF and Fig. 12 compares modeled nighttime vertical profiles of wind and temperature at IAP with observed profiles from a radiosounding at 0300 UTC. The lidar observations indicate two separate flow layers before sunrise. A westerly down-valley flow occurs between 200 and 800 m a.g.l., which lasts until about 0800 UTC and is capped by an easterly flow aloft. The radiosounding at 0300 UTC indicates that this easterly flow layer extends to about 2000 m a.g.l. (Fig. 12). Wind speeds in the lowest 200 m a.g.l. are very low, similar to what has already been seen for the surface winds (Fig. 11). Around 1000 UTC, the up-valley wind starts, reaching a depth of approximately 1000 m. The up-valley flow persists until 1600 UTC, when the flow changes briefly to a northerly direction, which may be caused by the winds down the mountain sidewall north of Innsbruck. The 2-h period of northerly winds ends with an abrupt change to a westerly down-valley direction. This down-valley flow has a pronounced jet profile, with the highest wind speeds of up to 7.5 m s$^{-1}$ around 200 m a.g.l. and weak winds near the surface.

The modeled wind direction in GRAMM-SCI deviates from the observations in the layer immediately above the down-valley flow, where easterly winds are observed, but the model captures the wind direction well above 1500 m a.s.l (Fig. 12). It produces, however, a strong easterly flow of more than 4 m s$^{-1}$ above the lowest approximately 900 m a.g.l., which turns to a more northerly direction during the day (Fig. 11). Note that WRF also exhibits a wind speed bias in the layer of north-easterly flow above 3500 m a.s.l.

Near the surface, a pronounced diurnal cycle is, however, simulated by GRAMM-SCI, with a 200-m deep westerly down-valley flow from midnight until around 0800 UTC. The down-valley wind system is replaced by an up-valley wind, which transitions into the northeasterly flow aloft and which has an intensity of about 4 to 5 m s$^{-1}$ consistent with the lidar observations. This system persists till 1700 UTC, when the down-valley wind develops again near the ground, reaching a depth of about 400 m around 2000 UTC. The down-valley winds are, however, slowly eroded until midnight by northerly winds aloft. Overall, GRAMM-SCI captures the observed transitions from down- to up-valley flows and vice-versa in the lowest few hundred metres.

WRF produces a very different flow behaviour. First, the down-valley wind system is deeper than in GRAMM-SCI, reaching up to around 600 m a.g.l. and thus closer to the lidar observations. At IAP, WRF does, however, not produce a westerly down-valley flow at all, but rather an easterly wind all the way down to the surface (Fig. 12). The transition from down- to up-valley winds occurs between 0600 to 1200 UTC, with very weak wind speeds. Later, a 1000-m deep layer of north-easterly up-valley flow develops around 1300 UTC. The late afternoon transition takes place around 1800 UTC, similar to GRAMM-SCI and the lidar observations. While the observations indicate a pronounced westerly down-valley direction in the evening, WRF shows a more south-southwesterly flow near the surface, which originates over the Stubai Alps (Fig. 9) and seems to cause a deflection of the down-valley flow over Innsbruck. GRAMM-SCI computes westerly winds and northerly winds above, which start to erode the down-valley flow until midnight.

The temperature structure in WRF, at 0300 UTC, is in good agreement with the observations above 1300 m a.g.l. Below, WRF is a few degrees too warm as it does not capture the shallow nearly isothermal layer at around 1200 m. Similarly, it does reproduce a layer of increased stability near the surface, but the stability is too low compared to the observations. GRAMM-SCI, on the other hand, has a warm bias throughout the valley atmosphere, up to 3000 m, with a deeper, 500-m thick inversion layer.

## Discussion

A GRAMM-SCI simulation was run for the Inn Valley with three nested domains and a 1-km horizontal grid spacing in the innermost domain using ERA5 data as initial and boundary conditions. Two sensitivity simulations were designed to further test the recently added model features and the sensitivity to the initial soil-moisture fields: (i) *GRAMM-dry* with initial soil moisture fields adjusted to better fit the measured values at the valley floor and (ii) *GRAMM-forest* without the above modifications to the land-surface model over forested areas. A WRF simulation was used as a benchmark for evaluating the GRAMM-SCI simulations. The selected case study was a typical undisturbed valley-wind day during summer convective conditions. No clouds were present and the surface-energy budget was strongly influenced by spatial heterogeneities in the terrain and land-use.

Both models generally overestimate the sum of the sensible and latent heat fluxes and do not capture the observed partitioning of the available energy into sensible and latent heat fluxes, with the exact partitioning varying, however, from site to site. For example, at the valley-floor site VF0, both models reproduce the observed latent heat flux relatively well, but WRF and the two GRAMM-SCI sensitivity runs overestimate the sensible heat flux. At the north-facing slope site NF27, on the other hand, the models overestimate the latent heat flux, but also the sensible heat flux to a lesser degree, with WRF agreeing best with the observed sensible heat flux. The partitioning of the fluxes at the second north-facing slope site NF10 is very similar to that at NF27 (not shown). The observed partitioning into sensible and latent heat fluxes changes, however, also throughout the day, which is reflected in a continuous decrease of the Bowen ratio between sunrise and sunset. This decrease in Bowen ratio is the result of differences in the timing of the maxima in the sensible and latent heat fluxes. In the Inn Valley, the sensible heat flux peaks mostly in the morning, while the latent heat flux peaks only in the afternoon (Lehner et al. 2021), which is also observed on the selected case study day. WRF and the GRAMM-SCI sensitivity runs, however, do not capture this shift in the respective peaks well, but tend to produce them close to each other and close to the time of the peak in shortwave incoming radiation. The result is that the Bowen ratio remains relatively constant throughout the day, with values below the observed ones in the morning and values higher than the observed one in the afternoon. However, not only do the models generally not capture the correct timing of the turbulent fluxes, but they also miss the timing in the ground heat flux, producing its peak in the morning several hours before the observed peak in the afternoon. With WRF performing only marginally better than GRAMM-SCI in capturing the timing of sensible, latent, and ground heat fluxes, clearly more work is needed in the modeling community to improve the surface-energy budget in mesoscale models.

An overestimation of turbulent fluxes has also been found in other studies (e.g., Sun et al. 2017). One problem when comparing modeled sensible and latent heat fluxes with observational data is that the observed surface-energy budget is rarely closed (Mauder et al. 2020), whereas it is closed by definition in numerical models. For example, for the Inn Valley, Lehner et al. (2021) have shown that the observed residual in the surface-energy budget can reach the same magnitude as the sensible heat flux. In this study, we have therefore introduced a new metric to evaluate the model performance by comparing the sum of the turbulent heat flux, latent heat flux, and ground heat flux biases in the model with the observed residual. This comparison is based on the assumption that the model reproduces the measured net radiation correctly. This assumption, however, turned out to be crucial. If the radiation was modeled correctly, as for example by WRF for the valley-floor site VF0, the total difference between modeled and observed fluxes matched the observed surface-energy budget underclosure. In the absence of correct radiation fields, it is difficult to establish the cause of the resulting differences in magnitude and timing between the observed residual and the model bias. While this metric thus seems to be a useful approach in evaluating model performance in simulating surface sensible and latent heat fluxes, it heavily depends on the modeled net radiation.

Parameterizing surface turbulent fluxes is challenging over complex terrain, including mountain topography and land-cover heterogeneity (Bou-Zeid et al. 2020). Similarity relations derived from observations over flat and homogeneous terrain are not necessarily applicable (Sfyri et al. 2018). Parameterizing evapotranspiration is further complicated by non-saturated soils (Cuxart and Boone 2020; Grachev et al. 2022). Generally, wet conditions also lead to bigger deviation of parameterized LH from observation than dry conditions (Grachev et al. 2022). An overview of currently used parameterizations for evapotranspiration is given by Cuxart and Boone (2020), in particular Monin–Obukhov similarity theory (Monin and Obukhov 1954, MOST) and surface-energy balance based methods, such as the Penman-Monteith (Penman and Keen 1948; Monteith 1965) and Priestley-Taylor (Priestley and Taylor 1972) approaches.

Grachev et al. (2022) proposed a new hybrid-bulk approach that combines the Priestley-Taylor approach for the latent heat flux with MOST for the momentum flux and sensible heat flux. They based their work on existing algorithms developed for open ocean and sea ice and extended it to relatively simple, flat land. A problem with methods based on the surface-energy balance is that observed residuals are oftentimes large, as in our case study. Progress on the applicability of MOST to complex terrain has been made recently through the consideration of turbulence anisotropy (e.g. Stiperski and Calaf 2022).

Despite the above deficiencies in reproducing the surface-energy budget correctly, both GRAMM-SCI and WRF capture the main flow characteristics relatively well. The models reproduce the weak wind speeds at the valley floor during nighttime, the daytime and evening transitions to an up-valley and back to a down-valley flow, respectively, and the complex interactions between valley and slope winds at the sidewall sites. The magnitude of the daytime up-valley winds at the valley floor and upslope winds at one of the north-facing slope sites is, however, underestimated by both models. The underestimation of the up-valley wind speed in WRF could be shown to be related to an underestimation of the horizontal pressure gradient between the centre of the valley and the valley entrance. Further analysis is needed to determine the exact causes, for example, whether the underestimation is related to inadequate grid resolution as has been shown in Schmidli et al. (2018) or to deficiencies in the valley’s energy budget, related to either incorrect representation of the surface fluxes or wrong land-use.

Previous studies evaluating GRAMM-SCI have shown that the model tends to overestimate nighttime katabatic winds (Oettl 2023). The modifications to the surface-layer scheme over forested areas described in the present study were designed to alleviate this problem. The comparison of two model simulations with (*GRAMM-base*) and without (*GRAMM-forest*) these new modifications shows that they are effective in reducing nighttime katabatic wind speeds at grid cells with significant forest cover (NF10 and NF27), resulting in wind speeds that agree better with observed values. Not only wind speeds are reduced, but also TKE and turbulent heat, moisture, and momentum fluxes. It has to be mentioned, however, that the results have not been compared with actual forest stations yet, since both NF10 and NF27 are located over grassland, even though forests are within a few hundred metres of the sites. Flux footprint calculations by Lehner et al. (2021) have, however, shown that the forested areas have very little impact on the measured turbulent fluxes.

The modifications to the surface-layer model in GRAMM-SCI act as a momentum sink over forested areas, reducing the nighttime overestimation of near-surface wind speeds, but they do not remove the overestimation of turbulent fluxes completely, which occurs even over non-forested areas. For example, the nighttime sensible heat fluxes are overestimated by about 25 W m$^{-2}$ at the valley floor in all three GRAMM-SCI simulations.

As mentioned above, both NF10 and NF27 are located over grassland, while the nearest grid cells in GRAMM-SCI have a forest cover of 57% and 79%, respectively. In WRF, which takes only the dominant land-use class within its $1\times 1$ km$^{-2}$ grid cell into account, the vegetation in the nearest grid cell to NF27 is equally classified as forest. These discrepancies between the model land-use and the real land-use complicate the model evaluation, since it is not clear if the model reproduces the observations for the right reason. At NF27, for example, *GRAMM-forest* without the reduced emissivity, reproduces the observed longwave net radiation significantly better than *GRAMM-base*, with a performance very similar to WRF. In this case, the supposed improvement to the surface-layer model in *GRAMM-base* is thus detrimental at first sight because the measurements are not taken within a forest and the simulation of below-canopy longwave enhancement is thus not improving the agreement with the observations. Similarly, both models strongly overestimate the latent heat flux at the south-facing site SF8 and at the same time underestimate the sensible heat flux. This site is located at the edge of a large concrete parking lot and helicopter landing zone, which thus reduces evaporation and increases the Bowen ratio, which cannot be expected to be captured by the models if the respective grid cells are covered by vegetation instead of impermeable surfaces.

While GRAMM-SCI reproduces the characteristics of the near-surface atmosphere reasonably well, it clearly has problems at upper part of the valley atmosphere, where it overestimates the wind speed by up to a few $m~s^{-1}$ and produces a warm bias of a few degrees, resulting in a too strong and deep surface inversion during nighttime. The reason for this deficiency is not entirely clear at the moment, in particular since it does not occur for other simulated case studies (Giovannini et al. 2023) and is thus not related to a systematic bias in the model.

## Conclusions

GRAMM-SCI (Graz Mesoscale Model-Scientific), a new branch of the mesoscale GRAMM open source model, has been evaluated against observational data over the complex terrain of the Inn Valley, Austria, together with the state-of-the-art WRF model. The evaluation focused in particular on the surface-energy budget and the wind field at different locations in the valley. Observational data came mainly from four eddy-covariance stations of the i-Box network (Rotach et al. 2017), for which a large dependence of the radiative and turbulent fluxes on local terrain and land-use characteristics has been shown before (Lehner et al. 2021). The goal of this study was to determine whether current mesoscale models of different complexity can reproduce the magnitudes, diurnal cycles, and spatial variability of the surface-energy budget components. Recent updates to GRAMM-SCI include the implementation of a PBL scheme using a prognostic TKE equation and modifications to the land-surface scheme for forested areas. The latter include specifically a new drag law in the momentum equations, an upper threshold for the roughness length to avoid unrealistically large turbulent fluxes, and an artificial decrease of the surface emissivity to mimic the below-canopy longwave enhancement effect of forests.

Overall, GRAMM-SCI has been shown to reproduce the daytime surface-energy budget and surface wind fields similarly well as WRF. Because of its design and associated modeling strategy, the model can be run with a small domain comparing to WRF. Because of that and mostly simpler physics parameterizations, it is generally faster and computationally less expensive.

The present study has also highlighted several general deficiencies of mesoscale models in simulating the surface-energy budget components, which were seen in both GRAMM-SCI and WRF:

$$\diamond $$
The models generally overestimate the daytime turbulent heat fluxes. While the surface-energy budget is closed in the models, large residuals are oftentimes observed, for example in the case studies presented here. It could be shown that if the model simulates the surface radiation correctly, the overestimation of the fluxes can indeed be explained by the observed residual.
$$\diamond $$
The diurnal cycles of the observed surface-energy budget in the Inn Valley are strongly impacted by the valley-wind circulation at the valley floor, but this impact is not captured well by the model. While the observed sensible heat flux reaches its maximum typically around solar noon and before the latent heat flux, resulting in a continuous decrease of the Bowen ratio throughout the day, the models do not reproduce the early and fast decrease of the sensible heat flux well, resulting in a comparatively small decrease of the Bowen ratio.
$$\diamond $$
The model evaluation with observations is strongly hampered by the difference between the model terrain and land-use on the one hand the and actual terrain and vegetation on the other hand, which means that the model grid cell is not necessarily representative of the measurement location.

These point to the fact that more studies are necessary to evaluate models over different types of terrain and land-cover characteristics and to identify errors in the model parameterizations, which will eventually lead to model improvements. For example, current land-surface models do not include the effect of horizontal fluxes which can, however, be important over complex terrain.

Full surface-energy balance stations are, however, relatively sparse, in particular over complex terrain. Hence, we do not only need more modeling studies, but also more dedicated measurements in particular over complex terrain. Recent field campaigns over heterogeneous land cover, for example, LIAISE (Land surface Interactions with the Atmosphere over the Iberian Semi-arid Environment; Mangan et al. 2023) and MOSAI (Model and Observation for Surface Atmosphere Interactions; Lohou et al. 2022), and the upcoming TEAMx program (Multi-Scale Transport and Exchange Processes in the Atmosphere over Mountains-Program and Experiment; Rotach et al. 2022) in highly complex, mountainous terrain will provide important observational data for future model evaluation studies. Already ongoing model intercomparison studies in the framework of TEAMx, in which both WRF and GRAMM-SCI are participating, will also shed further light on the performance of mesoscale models in mountainous areas and help to identify and hopefully improve existing deficiencies.

## Data Availability

The namelists and outputs from WRF and GRAMM-SCI models, the data from observations and the post-processing codes are available on request to the authors.

## Footnotes

1 https://www.ecmwf.int/en/forecasts/datasets/reanalysis-datasets/era5.

2 https://land.copernicus.eu/pan-european/corine-land-cover.
